# The Social Construction of Narratives and Arguments in Animal Welfare Discourse and Debate

**DOI:** 10.3390/ani12192582

**Published:** 2022-09-27

**Authors:** Mukhtar Muhammad, Jessica E. Stokes, Lisa Morgans, Louise Manning

**Affiliations:** 1 Department of Agriculture Food and Environment, Royal Agricultural University, Cirencester GL7 6JS, UK; 2 Innovation for Agriculture, Kenilworth, Warwickshire CV8 2LZ, UK; 3 Lincoln Institute for Agri-Food Technology, University of Lincoln, Riseholme Park, Lincoln LN2 2LG, UK

**Keywords:** animal welfare, argument, dialogue, stakeholder, sheep, discourse, narrative

## Abstract

**Simple Summary:**

Animal welfare is a public good that is extremely important to stakeholders, who can hold conflicting values and viewpoints, on what animal welfare is, and how a good life is achieved. Various stakeholder groups tend to signal different problems, or problematize specific aspects of farm animal welfare, and propose different actions or interventions within food supply chains. In the paper we explore the contribution of narrative and argumentative discourse to the social construction and framing of animal welfare and its implications. Our findings demonstrate the contestation within the stakeholder discourse around animal welfare and farm animals. We demonstrate how performance-related perspectives are rooted in value-laden language within narratives that shape the discourse regarding notions of good and bad welfare; or positive or negative welfare. We suggest that future research should examine in more depth the socially constructed language, dialogues and discourses expressed among and between stakeholders in order to embody the breadth, depth, and understanding of the multiple meanings of farm animal welfare, and the emergent positioning of positive welfare for farm animals as well as how to achieve a good life in practice. The novel contribution of this review is the application of an explanatory word-language-discourse-person-situation-environment framework in this specific context to inform future research on animal welfare discourse analysis.

**Abstract:**

Stakeholders can hold conflicting values and viewpoints, on what animal welfare is and how a good life is achieved and can signal different problems, or problematize specific aspects of farm animal welfare, and propose different actions or interventions within food supply chains. The aim of the study is to explore the contribution of narrative and argumentative discourse to the social construction and framing of animal welfare and its implications. The methodological approach in this research is composed of two phases with phase 1 being the foundational structured literature search in both academic and grey literature. Phase 2 was the analysis of the secondary data from the literature review to develop a synthesized iterative paper and in doing so develop a typology of five narratives: the ‘farming as a business’ narrative, the ‘religion-based’ narrative, the ‘research, legislative and political based narrative’, the ‘higher welfare’ narrative, and the “animal rights/power-based” narrative. Our findings demonstrate the contestation within the stakeholder discourse of the articulation of why farm animals should have a good life. Performance-related perspectives are rooted in the value-laden language and narratives that shape the arguments regarding notions of good and bad welfare; the emergent positioning of positive welfare for farm animals as well as how to achieve a good life in practice. The novel contribution of this review is the application of an explanatory word-language-discourse-person-situation-environment framework in this specific context to inform future research on animal welfare discourse analysis.

## 1. Introduction

A discourse is simply, communication in either the written or spoken word about a specific topic, i.e., the word as a single unit lies at the heart of the discourse and how the words are arranged or structured in a given sequence forms language. A discourse is developed from a constructed arrangement of language, and can include a monologue, i.e., a one-way, report, statement or commentary and dialogues which are multiple-way communication or conversations. Discourse can be considered for its linguistic content, the subject matter and its linguistic form, namely its cohesion and structuring, as well as the socially framed meanings. These are often positioned in the discourse through rhetoric devices in the argumentative organization of the text [1].

The term “dialogue” derives from two words in classical Greek, “dia” meaning “through” and “logos” meaning “word” [2]. The direct interpretation of the term implies that engaging in dialogue creates communication, harmony and understanding. Dialogues are “key to our inspiration and to our capacities to sort out ethical dilemmas and the multiple meanings that confront us as we continue our inquiries into human experience” [3] (p. 51). Dialogues between stakeholders in this context have been described alternatively as intensified [4]; institutionalized [5]; collaborative, cogenerative, consensus driven [6,7,8]; mutually beneficial [9]; constructive [8]; pluralistic [10]; persuasive [11]; synchronous or asynchronous [11]; internal or external [12]; and surface or deep [13]. These descriptions of dialogue demonstrate the multifaceted aspects of engaging in dialogue (Table 1).

Dialogue may take many forms, such as persuasion, deliberation, eristic, negotiation, inquiry (scientific), and inquiry (philosophical). Indeed, based on the extant literature, citizens’ and consumers’ dialogue can be classified from a philosophical and ethical stance [14,15]; and driven by cognitive appraisal of what individuals believe is right or wrong, which may, or may not be scientifically supported. Persuasion uses rhetoric strategies to influence others and is an element of overall communication with both analytical approaches (focusing on logic), and dialectic (debating a point or issue) elements [16].

Dialogue may be primary or secondary dialogue. Primary (or direct) dialogue forms the ‘visible’ argument and or communications of an organization and is embedded in external corporate documents such as annual reports and websites [16,17]. Primary dialogue is the actual words or language that are used in a conversation between two or more individuals. Secondary dialogue represents the “silent” or “shadow” internalized discourses which may not be visible but are perceived to be embedded or inferred in primary dialogues [16]. Simply put, primary dialogue is what we say (the words and structured content of the language), while the secondary dialogue is not what is said ‘but implied’ in the language which is used or not used. Considering this statement, ‘this sheep flock has a very high level of lameness’ could through the secondary dialogue be taken to have the inferred meaning that “you are a bad farmer”. This interaction between primary and secondary dialogue through discourse can operate at the discussion level between individual farmers, advisors, certification bodies, standard owners, and service providers and in the written communication. However, investigations into this research area in terms of the social construction of discourse and its meaning(s) are currently lacking.

Animal welfare is a public good that is extremely important to multiple stakeholders. Stakeholders can concur or alternatively hold conflicting values and viewpoints, on what animal welfare is, and how good welfare, in particular, is achieved. Various stakeholder groups tend to signal different problems and/or problematize specific aspects of animal welfare and propose alternative solutions [14,18]. Their conceptualizations of animal welfare are based on specific and various frames of reference [18,19]. These often reductionist framings help to make sense of complex realities: providing a perspective to structure knowledge, position experiences, and to judge and respond to issues [18,20].

Within the framing processes, and through social construction, different groups compete against each other to provide the primary discourse, as well as engender public recognition and support [21], through articulating a range of argumentative positions within the given discourse [22]. Peoples’ adoption of a specific animal welfare frame, discourse or given perception, depends on the role an individual has, or the organization they represent, and as a result, this framing may vary over time and place, i.e., the situation and the environment [23] for example, where and to whom they are speaking to about animal welfare [24]. An example of how this can be seen in practice is when a farmer is at work with their livestock compared to their pets at home; their perceptions of acceptable standards of welfare in these two situations may vary. For this reason, among others, recurring aspects in the collective animal welfare discourse, such as what good welfare practice is and what it entails, remain contested due to different stakeholders’ frames and understanding. 

There are many studies examining stakeholders’ framing and perceptions of animal welfare [24,25,26,27,28]. Gender and extended society, it is suggested, has an impact on different perceptions of welfare issues and welfare indicators, as women and the public show more concern for animal welfare and painful husbandry practices compared to others [26]. Furthermore, in their multi-stakeholder research, Ref. [18] found that pig farmers show preference for a biological functioning approach to framing animal welfare, which emphasizes animals’ health, fertility, and productivity. In contrast, both animal scientists and urban citizens see pigs as natural living beings, which emphasizes the need for good mental welfare and the need for them to live in environments where they can behave naturally. Similarly, surveys in the United States (US) and the United Kingdom (UK) found that consumers view better living conditions for farm animals as very important for good animal welfare [29]. All these findings are relevant, but they are primarily attitude-based responses, and therefore do not explain the socio-constructive mechanisms through which these attitudes and perceptions are formed, and reformed.

The aim of the study is not to review societal understandings of good animal welfare per se (see [30]), but rather to explore the varied contribution from different stakeholders to the socially constructed narratives and argumentative discourse about animal welfare and its implications to add to our wider understanding of communication around animal welfare across the food production related discourse. This conceptual approach is informed by the work of [23,31,32] who examined the social construction of arguments more generally and also within the animal “rights” discourse. This critical review herein, considers how different stakeholders construct their perceptions of animal welfare based upon these contested narratives and arguments and how this informs the defining of good animal welfare, and a good life in the wider discourse. This review will identify the types of narrative that have emerged in this discourse around food production and in particular, notions of good animal welfare, and how this may relate to good husbandry and good agricultural practice. In this context, the ‘narrative’ is considered to be the stories told by humans which reflect their way of looking at the world [33]. The ‘argument’ is the set of statements or reasoning in support of a given viewpoint within the dialogue.

The paper is structured as follows: Section 1 is the introduction and Section 2 explores the narratives and arguments in support of good animal welfare. Section 3 considers the role of disclosure, strategic ambiguity and transparency within stakeholders’ dialogue and then how dialogue can be used as a means to resolve contested positioning on animal welfare. Section 4 concludes the paper.

## 2. Arguments and Narratives in Support of Good Animal Welfare

Most consumers appear satisfied with the quality and safety of food at point of sale but some remain skeptical about the management practices and associated standards used in food production [26,34]. Consumers are interested in knowing that producers care about and provide for the welfare of animals during the production process [28]. This is more evident if stakeholders are responsible for sourcing meat from high welfare farms. However, it is widely understood that consumers’ concerns are not equally distributed across the different farm species, nor is there consistency in consumers’ willingness to pay to enhance animal welfare [28]. Therefore, it becomes imperative to understand how public perceptions of farm animals’ welfare are formed, are changing, as well as how stakeholders, including consumers, have adopted non-market strategies such as arguments and dialogues to drive improvement in farm animal welfare collectively [35].

There are various forms of argumentation including an *argument-to-learn* mindset and an *argument-to-win* mindset [36,37]. In the argument-to-learn mindset, each person genuinely attempts to discover more about the issue under discussion and to arrive at a more accurate answer. Dutilh [37] describes argumentation from this viewpoint as an epistemic practice, in increasing knowledge, or in fostering understanding. In contrast, the argument-to-win narrative is “simply to score points”, with each participant attempting to emerge victorious over the other and having no interest in learning new information or modifying their views [37]. In the debate over good animal welfare practices, there is much interplay between these arguments used by and among stakeholders. As an example, consumers/citizens can consider welfare improvement as a collective stakeholder responsibility. Collective responsibility associates both causal responsibility and blameworthiness on groups and locates the source of moral responsibility in the collective actions taken by these groups [38]. Therefore, consumers/citizens can use “prescriptive statements” (e.g., we want responsibly sourced meat) or ‘culpability language” (e.g., statements such as intensive farming is bad for welfare, or shearing is terrible for the animal) to hold stakeholders to accountability-based standards. With such arguments, consumers/citizens can hold farmers accountable for what they perceive is right and wrong in animal production, and what practices are adopted and implemented on their behalf.

Historically, the animal welfare debate has been driven by the value-driven concept [25,39], where contestation can then occur. Value-driven conflict primarily arises not because one specific value is perceived as correct or incorrect, but instead the order of priority given to a set of values, e.g., for a good life for farm animals varies between stakeholders [18,40]. In this regard, the notion of a good life for farm animals moves beyond notions of what good welfare is, because defining a good life also encompasses the aspect of sentience [41]. Citizens and animal rights organizations can perceive animal welfare from a differentiated philosophical, ethical and/or scientific stance [14]. Animal welfare organizations’ campaigns can use the media to illustrate specific aspects of farm animal management to build opposition to a particular farming practice, or system, lobbying and putting pressure on retailers to influence animal husbandry and animal welfare [15]. Animal sentience considers that animals experience both positive and negative emotions such as joy, pleasure, pain, and discomfort [42]. To achieve their aim, animal welfare organizations voice their concern using words related to animal sentience like “pleasure”, “pain”, “suffering”, and “happiness”. Scientists and veterinarians themselves use these words, for example “comfort” and “pleasure” forming the basis of scientific assessments of positive welfare [39]. Thus, it is the value placed on animal care and treatment that shapes discourses and narratives of what good welfare is and ultimately a good life. 

Value-laden approaches can be condensed and classified from either ethical (moral) or utility perspectives [14,43]. The ethical (moral) premise firmly holds that humans have a responsibility or duty to care for animals especially in prevent suffering [44]. In this moral premise, it is argued that animal welfare should be considered as intrinsically important [45]. Consequently, animals suffering, fear, pain, or stress may be defined as experiencing negative or poor welfare, and such experiences should be minimized or eradicated for the animals. Clearly, ethic arguments establish binary classifications of good and bad welfare, positive and negative language, although the impacts of this choice of language on farmers’ decision-making and actions with regard to animal welfare have rarely been explored. 

On the other hand, the utility notion, denoted by anthropocentric conservation, holds that ‘ill-treating an animal does not infringe any morally important interests the animals themselves possess, but infringes the interests of other humans in the process’ [44] (p. 52). This postulation excludes notions of sentience and implies that as the intrinsic, innate value of animals is hard to observe, so their care and protection rest entirely on the vested interests of humans [44]. A major argument in favor of anthropocentric theory is that it is difficult to capture in economic terms the value of pleasure, happiness or comfort in animals. Therefore, anthropocentric welfarism posits that only the welfare of humans counts intrinsically as opposed to moral disposition [44]. Some principles within utility arguments are useful when describing discourses and narratives of key industry stakeholders.

To illustrate how these values are reflected in stakeholders’ argumentative discourses regarding animal welfare, and to provide insight into the interplay by, and between, animal welfare argumentative discourses, we position five narratives in this context namely the ‘farming as a business’ narrative, the ‘religion-based’ narrative, the ‘research, legislative and political based narrative’, the ‘higher welfare’ narrative, and the “animal rights/power-based” narrative. Examples are provided to give context to some of the narratives described.

### 2.1. The Farming as a Business Narrative

The utilitarian approach implies that animals’ intrinsic, innate value is hard to observe; thus, their care and protection rest entirely on the vested interests of humans [45]. Considering this assumption, farmers often engage in discourse related to agro-industrial production to achieve economic gains or mitigate potential losses. Farmers have been found to link “good welfare” with productivity and profitability in their arguments [22]. Therefore, statements such as “producing more with fewer resources” is common language at the farm level, derived from discussions centered around farm efficiency and sustainability (economic, environmental and social aspects). Such established narratives and arguments may focus on intensifying animal and food production, with animals perceived in terms of utility as a production unit to deliver broader socio-economic outcomes. 

To meet global food demand for a rising human population, animal production and its intensification are positioned in narratives as being essential, contributing significantly to ensuring food security [46]. Management strategies such as genetics and selective breeding to produce the most optimal, efficient animals can be seen within this intensification narrative. These strategies provide an opportunity to reduce economic costs per production unit, which can be passed through the supply chain and to the consumer. The farming as a business narrative also focuses on how such management practices improve animal performance and the economic efficiencies of farming operations. However, the “producing more with fewer resources” argument in livestock farming may not consider the health, welfare, or sentience of farm animals. Moreover, increased livestock production may result in more greenhouse gas emissions (GHGEs), e.g., carbon dioxide or ammonia, that could promote climate change and harm farm animals’ welfare in the future. In this situation, efforts to estimate the level of GHGEs of a variety of economic activities associated with farm animals, as well as investigating sustainable mitigation strategies including promoting farm animal welfare is important [47,48]. 

For animals to perform productively and profitably for the farmer, they need to stay fit and healthy. In this regard, farmers tend to give priority to the prevention of disease and injury, as well as ensuring access to food, water, shelter, and other necessities of life, concerns that could be summed up as being focused on the essential health and functioning of the animals [24,40,49]. Specifically, however, the narrative herein is one of cost-utility and cost-effectiveness, especially around the management of zoonoses, which impact on both animal and public health [50].

### 2.2. The Religion-Based Narrative

In philosophy, the major world views believed to have influenced animal welfare, especially in texts and writings, are the classical Greek thoughts and Judeo-Christian perspectives. These views are believed to have contributed to inequalities regarding human and animal rights, paving the way for a long history of human privilege over animals [51,52]. Judeo-Christian perspectives incline towards the notion that humans have dominion over animals, and this view remains one of the most populous beliefs among many [53,54]. According to biblical sources, man’s superiority over animals comes with a moral obligation to the animal [53,54]. These moral obligations interface with the Five Freedoms’ principle (for example, freedom from hunger and thirst (see [55]) as well as underpinning an ethics focused argument. 

In Islam, there is a strong emphasis placed on the importance of balancing the narratives of utility, ethics, and power. From the Islamic perspective, animals were created for humankind’s benefit and use. Humans are prohibited from using farm animals in ways that are not prescribed. In the Quran, it states, “And the grazing livestock He has created for you; in them is warmth and [numerous] benefits, and from them you eat. And for you in them is [the enjoyment of] beauty when you bring them in [for the evening] and when you send them out [to pasture]. And they carry your loads to a land you could not have reached except with difficulty to yourselves. Indeed, your Lord is Kind and Merciful. And [He created] the horses, mules, and donkeys for you to ride and [as] adornment. And He creates that which you do not know.” (Quran 16: verse 5–8). It can be drawn from this textual evidence that naturalness plays a key part in the perception of farm animals’ lives in the Quran. This aligns with the naturalness definition of animal welfare, which plays an essential role in facilitating the behavior of farmed animals, such as sheep, as they graze in open environments, i.e., the freedom to express normal behavior, as well as the importance of protection and shelter at night. Existing research in the literature links the Quran to animal welfare [56,57]. These narratives frame notions of compassion, naturalness, and freedom from pain and cruelty within Islamic teaching which demonstrate the arguments of reaching good welfare states through minimizing negative welfare. However, practice may not always reflect the religious narrative, and research advocates for the sensitization of religious followers to the teachings of animal welfare in the Quran and the Hadiths [58]. Religion-based narratives can therefore influence how animals are socially, culturally, and politically viewed and ideally treated in human society. These religion-based narratives can be formalized into industry standards and codes for example the halal certification standards implemented by the Malaysian organization Jabatan Kemajuan Islam Malaysia (JAKIM) [59].

### 2.3. The Research, Legislative and Political Narrative

Political narratives on animal welfare can be traced back to the 1960s where the perspective was to consider welfare predominantly from the angle of animal cruelty and suffering [60]. The then UK Government assembled an expert committee to investigate cruelty concerns raised about animals in intensive confinement systems. The Committee, compiled the Brambell Report, which criticized the state and health of farm animals confined in intensive systems [61]. The report maintained that farm animals produced in such systems demonstrated signs of frustration, distress, and pain, as well as being capable of emotional states such as fear, pleasure, and happiness [50,61,62]. The Brambell Committee therefore recognized farm animals as sentient beings, the concept of which was enshrined in animal welfare as a key consideration for husbandry management systems [61].

The Brambell Committee report was also instrumental in developing the Five Freedoms model, which emphasizes the importance of eradicating negatives through good husbandry practices. Historically, the Five Freedoms has been used to assess animal welfare; however, it has been positioned as having some significant limitations. For example, the importance of physical and functional aspects of animal welfare (malnutrition, disease, and injury) are not distinguished from the affective aspects (thirst, hunger, discomfort, pain, fear, and distress) [63]. Moreover, Ref. [64] highlighted that the Five Freedoms were primarily concerned with eradicating negative welfare aspects of behavior, while only freedom to expressing natural behavior promoted positive states of being. As a result, there has been a shift in animal welfare science thinking, and it became increasingly important to consider positive experiences when providing a holistic understanding of animal welfare. In 2015, the Five Domains based on physiological aspects to account for the positive states of the animal were introduced [65]. Adding the ‘mental experience’ element of animal welfare emphasized the importance of providing animals with positive experiences in the Five Domains, such as comfort, pleasure, confidence, and enjoyment [65]. As part of further revision of the Five Domains, stockmanship skills and qualities have been included, emphasizing the importance of human intervention in ensuring positive outcomes [66].

The work of [67] also unifies aspects of subjective feelings, health and diseases, and natural behavior in outdoor environments (naturalness) as being complementary, not antagonistic, concepts. Affective states and biological functioning are now incorporated into animal science assessments since they both take a physiological approach and complement one another in welfare assessments [68]. For instance, a highly managed animal may suffer poor welfare if environmental challenges overwhelm its evolved coping strategies for example, in high temperatures if behavioral and physiological adaptations fail to dissipate heat sufficiently [69]. The concept of naturalness captures common behaviors that are either reflecting approval or disapproval, but naturalness is not easily compatible with concern for animals’ affective elements, suggesting that they remain antagonistic to some extent [70]. So far, naturalness has only been used as a baseline for positive welfare assessment [65]. 

The Brambell Report drove the legislative narrative for the protection and enhancement of animal welfare. Legislation such as the UK Agriculture (Miscellaneous Provisions) Act in 1968 was one of the first regulatory Acts which banned livestock managers from subjecting farm animals to a traumatic experience [61,62]. Under this Act, the Codes of Recommendation for Animal Welfare, produced for the major livestock species, including cattle, pigs, sheep, and poultry birds were formed [61,71]. These welfare codes explicitly contain policies recommended from the Brambell Report on safeguarding the welfare of farm animals under various management practices. The ‘Codes’ are developed as guidelines for how farmers apply the law but have no legal authority on their own, but noncompliance is seen as evidence of poor welfare standards [71] and can be used as evidence for prosecution. The ‘Codes’ are also embedded in major UK farm assurance schemes, such as Red Tractor [72], Royal Society for the Protection of Animals (RSPCA) Assured [73] and Soil Association (organic) standards [74]. These standards within farm assurance schemes are often set at a higher level than regulatory standards, especially if scheme membership is used as a market access tool for entry to specific retail or foodservice markets. Key UK legislation enacting the rules and regulations of safeguarding animal welfare includes the Animal Welfare Act (2006). Other examples include the Australian Animal Welfare Standards and Guidelines [75], and the World Organization for Animal Health Codes and Manuals formerly the Office International des Epizooties or OIE [76]. In summary, the regulatory and policy driven narrative positions that legislation is designed to safeguard the health and welfare of farm animals and to minimize negative welfare aspects, but in recent years, policy is moving in the direction of incentivizing positive welfare towards providing a good life [77]. 

### 2.4. Higher Welfare Narrative

The utility concept considers that animal production systems are unproductive, uneconomic, or unviable if the consumer is not willing to pay more for non-utility aspects of animal welfare. The literature suggests that this utility narrative has driven commodification of animals [78,79]. Market segmentation means many retailers work with suppliers and set farm animal welfare standards within their contracts and conduct regular audits or inspections of suppliers’ premises and practices based on these standards [80]. Nevertheless, most of these standards and measures are understood to be primarily aimed at delivering good welfare through minimizing negative welfare rather than promoting positive welfare towards achieving a ‘good’ life. 

For example, the utility attributes of standard and higher welfare eggs have been examined, and how they relate to the willingness to pay for them. Additional information was found to significantly increase intention to purchase higher welfare rather than conventional welfare products [42]. Examples of higher welfare standards include the New Zealand Society for the Prevention of Cruelty to Animals (SPCA) certified standards [81] derived from the Five Domains of Animal Welfare and from France, the Label Rouge standards [82]. Providing information and not just promoting heuristic processing enables consumers to better align their values or concerns with their purchasing intentions [83]. Public concerns about farm animal production methods do not always correspond to purchasing and consumption patterns, with sales of higher welfare products reported to be far lower than levels of reported concern [26]. This has widely been associated with cognitive dissonance, where an individual’s actions contradict their beliefs or values, and as a result discrepancies occur between an individual’s perceived role as a citizen and their actions as a consumer [28,34,84].

According to the Food Ethics Council [85], citizens can positively influence how food is produced, distributed, and consumed. Citizens are vocal in their opinions on animal welfare and look to legislation, government and other stakeholders to improve and raise standards [49]. People can cause a significant shift when they act as citizens rather than consumers but acting as consumers rather than citizens creates cognitive dissonance between prosocial attitudes and non-prosocial behaviors. Therefore, the consumers’ role can be confined to choosing between products and services and not necessarily involve emotional participation and influencing food systems through purchasing behavior. Consumers in this context are affected by what they can afford, and their income can inform their preferences [39,86,87]. Bansback [87] (p. 6) argues that:

“*Price factors are still the most important determinants of meat consumption … the ability of the industry to reduce its costs relative to other competitors is getting more limited. Income effects … are also of less importance in influencing demand (however) consumer attitude/preference issues are growing in importance.*”

Therefore, not all consumers may consider, or be able to financially consider, animal welfare at the point of purchase. A survey conducted as part of the Welfare Quality Project in the UK and six other countries supports this argument [28,88]. According to the survey, 73% of respondents are interested in animal welfare. However, only 39% of respondents consider welfare when purchasing meat, others adopting dissonance strategies to enable their guilt-free meat consumption [26,89]. Considering this apparent disconnect, and “unwillingness to pay” mediating between citizens’ concerns, preferences, and consumers’ consumption, it becomes imperative to understand the unresolved issues emanating from often contested, even undisclosed animal welfare related narratives. It is our understanding that there have been less studies examining the influence of expressive or descriptive language (whether positive or negative) within narratives in the dissemination of information about animal welfare. It is possible that further exploration of this topic will contribute to a better understanding of the dynamics of consumer choices, and the impact that words, language and the monologue/dialogue within a specific narrative may have on those choices. 

### 2.5. The Animal Rights/Power-Based Narrative

Animal rights groups raise concerns with specific farm practices and systems, detailing the consequences for the animals’ welfare [90]. These stakeholders focus on inducing negative emotions using words like pain, fear or stress, to describe outcomes which can be caused by certain husbandry practices, justifying ethical claims against these practices. Animal rights groups focus on animals as sentient beings and will contest any practice that would subject farm animals to negative experiences. An example of a campaign that has utilized this narrative is the Plofkip campaign with chickens in the Netherlands in 2012 [91] which focused on ‘fast growing’ broilers. Thus, through their narrative animal rights groups challenge and reject the commodification of animals in society, condemning animal products such as meat, wool or fur because the animal may experience pain and other negative experiences during the production process. These organizations draw upon a combination of scientific, emotional, and ethical perspectives to criticize animal farming and farming practices, seeking to influence and change consumer acceptance that perpetuates these systems. In summary, the underlying argument of animal rights groups are that good quality of life is not afforded to farm animals, when sentience is violated by farming practices that subject animals to predominantly negative welfare experiences. 

The negative narrative of cruelty, abuse, trauma, or torture is powerful in terms of the descriptiveness or expressiveness of the language, leading to feelings of disbelief, sympathy, concern and even condemnation in the people (recipients) who interact with it. Thus, the language can be considered as powerful in terms of either its expressiveness (words used) and/or the narrative can express notions of being empowered (the human) versus unempowered (the animal) and as a result imply a power imbalance and notions of abuse of that power within the human–animal relationship [92]. Jennings et al., [93] describe this as ‘*the power of talk’*, i.e., the power of communication, or ‘*the power in talk’*, i.e., the power/powerlessness dynamics embodied in the language used in communication. Deetz et al., [94] (p. 32) position the power of talk:

*“we conceive of power neither as simply a possession of individuals nor a relationship between individuals, but rather as a structural quality of institutional life, which is chronically reproduced by the day to day communicative practices of its members”.*

and the power in talk,

*“Power is conceived as involving a relationship of autonomy and dependence between social actors or groups, then power is exercised in the context of a struggle between domination and resistance…… is conceived as the process through which competing interests exist interdependently, simultaneously vying for a privileged status in the whole constellation of interests that characterize institutional life”.*

These explanatory passages demonstrate how stakeholders compete for control and content of narratives and arguments adopted and based on power. Thus, societal processing of the narratives and arguments depends on the situational aspect (what it is about) and the level of cognitive engagement with the discourse (deriving what it means). These two elements are influenced by the properties of the communication and the contextual cognitive assessment by the recipient. Media culture “helps shape everyday life, influencing how people think and behave, how they see themselves and other people, and how they construct their identities” [95] (p. 2). There are many public spaces where images of animal cruelty are placed, debated, and reproduced [96]. In these spaces, the media coverage typically describes cruel, animal-related food production, or ‘factory farming’, using the utility narrative describing animals as economically exploitable production units and commodities [87]. Power-based media narratives typically focus on ethical issues in agricultural production, particularly farm animal welfare [97]. Constructed arguments were used to question the activities and motives of the wool industry in countries like Australia, especially practices such as mulesing, leading to a decrease in sales of lamb and wool due to activism driving lower consumer demand [89]. Media representations of farm animal welfare issues are important because the media is a significant source of information for consumers/citizens, and the way that issues are represented, or framed suggests causes, solutions, or provides moral evaluations [97].

In summary, the typology of five narratives on farm animal welfare presented here reflect the social construction of varying perspectives on the purpose and treatment of animals in society. The social construction process enacted by different stakeholders is influenced by experience, background knowledge and cultural values [14,40,43]. This review has positioned language, narrative and arguments in the contested discourse of animal welfare. These narratives can be seen along a spectrum, from positioning animals as production commodities (farming as a business narrative), to humans having a moral obligation to protect animals and prevent cruelty (religious based narratives), welfare optimized within and through new farming systems (higher welfare narrative), to consideration of animals as sentient beings, capable of positive welfare experiences (research, legislative and political narrative), to reflecting how a good life can be achieved and rejecting the utility based concept of animals as units of food production (animal rights/power narrative). According to [22], these differences in the articulation of animal welfare stem from varied ideological positioning affecting the social construction and framing of these debated issues and presenting a barrier to effective communication and resolution of conflicts, particularly as livestock producers face increasing scrutiny by society. It is therefore important to bring actors together from across the ideological spectrum, to provide mutually beneficially insight and understanding, as a means of building and collaborating around common ground. The next section considers how dialogue can be used to resolve such contested positioning on animal welfare.

## 3. Dialogue as a Method of Resolving Contested Positions on Animal Welfare

Narratives considering what is good or bad animal welfare are framed from the argument-to-win perspective. This fixed argument is not effective in driving change as individuals only wish to affirm/reaffirm their own position. Furthermore, farmers can find it challenging to embrace prescriptive welfare frameworks imposed on them by the scientific community, or to comply with public concerns that are contrary to their own philosophy [98]. Indeed, scientific bodies and the public may not value the importance of tacit knowledge of aspects of animal welfare that can only be gained from personal experience with animals. There is also a lack of knowledge within the scientific community regarding the economic cost and other barriers to applying certain measures relative to their health and welfare benefits, which impedes their widespread implementation [98]. Therefore, engaging in two-way dialogue can be beneficial for understanding differences and resolving conflicts between stakeholders’ perspectives, and promoting different narratives and arguments on achieving good animal welfare [99]. Three communication strategies used in corporate communications reflect how corporate or organizational values are established via embedded strategy and the integration of stakeholder expectations [100,101]. This is important when considering a values-laden narrative.

*Information strategy* is where corporate strategy is developed in isolation, priorities set and then communicated as a monologue through corporate communications. This communication is one-way, e.g., press releases, or information statements [86]. In this form of communication, the expectations of interest groups or end users are not always overtly integrated into the corporate vision. An example of this form of dialogue includes the Animal Health and Welfare Statement by UK retailer Sainsburys [102]. Alternatively, stakeholders such as farmers, food companies and retail businesses can often use a monologue *response strategy* to deliver to stakeholder or consumer demands, i.e., a reactive rather than proactive unidirectional communication approach. Examples of response strategy communications are corporate social responsibility (CSR) reports or company annual reports [101]. These can be prompted internally or externally to the business and are crafted in terms of words, language and discourse especially with regard to target audience (s). Conversely, *involvement strategy* is where a dialogue takes place between stakeholders, e.g., via social media, online forms or meetings with interest groups, with implications for the definition/redefinition of normative standards or corporate values and associated CSR activities [101]. Involvement strategy is normally used by mediating bodies that aim to promote dialogue and understanding in the animal welfare debate seeking to protect consumer interests, whilst also listening to farmers and others to achieve a common objective. Advisors, certification bodies, standards owners, and service providers engaged with involvement strategies may work with a broader stakeholder base such as farmers, manufacturers, animal welfare groups and retailers and also mediate to protect consumers interest in animal welfare. An example of an international involvement strategy addressing animal welfare is the Better Chicken Commitment where over 200 food companies have now publicly committed in organizational statements to meet specific welfare standards within given timeframes [103]. 

### 3.1. Disclosure, Strategic Ambiguity, and Transparency

Welp et al., [104] define stakeholder dialogue in scientific research as structured communication processes that link scientists with societal actors, such as representatives of companies, non-governmental organizations (NGOs), governments, and the wider public. A key aspect of this definition is the linkages between scientific knowledge and local, tacit or indigenous knowledge. The intent of these collaborations is to generate new ideas and co-create new solutions to contentious issues. Thus, the main objectives of science-based stakeholder dialogues are to deepen the collective understanding of contentious issues, combine scientific knowledge with other indigenous sources, and to check the social relevance of concepts [104]. To support the implementation of good animal welfare practices, there has been a growing stakeholder alliance seeking animal welfare improvement initiatives through analyzing monologues as a form of disclosure. One of these alliances is the Business Benchmark on Farm Animal Welfare (BBFAW), a global measure of reporting corporate disclosure of farm animal welfare practices and management, and corporate performances [105]. Based on the degree and content of corporate disclosure, the framework evaluates how close, companies are to best practice in three areas: Management Commitment, Governance and Performance and Leadership and Innovation [106]. It allows investors, businesses, NGOs and other stakeholders to understand and inculcate reported corporate practices and performances on farm animal welfare and create a response narrative via the BBFAW with the aim of driving corporate improvements in the welfare of farm animals. Levy et al., [107] (p.389) assert that non-governmental organizations (NGOs):

*“Participated in a sequence of strategic interactions that ultimately resulted in substantial concessions by corporate actors, who adopted many of the elements of the discourse, business models, and governance structures proposed by NGOs.”*

However, it is important to note that this benchmark looks at reported (publicly disclosed) policies and protocols. Public disclosure means “most … {corporate} … statements are only relevant to stakeholders (such as retailers and food companies’) own brand product” [108] and may rarely provide actual or real-time information disclosing the spatial or temporal status of animal welfare under the responsibility of a given business or supply chain. For this reason, it is possible for stakeholders and consumers to perceive an organization’s degree of reporting as lacking transparency if either vague or ambiguous language is used or fully or partially information remains undisclosed. Consequently, this may give the impression that sensitive corporate information may be being withheld from the discourse provided to or with external stakeholders, providing concerns over greenwashing or ‘welfare washing’ [109].

Thus, self-reported publicly disclosed information is potentially a strategically ambiguous discourse that may lack content-related clarity and instead seeks to accomplish certain organizational goals. The rise of strategic ambiguity in these stakeholder communications means that an organization can promise a practice in the future or hint at contemporary practice without it necessarily being verified independently to ensure it has been adopted. Strategic ambiguity can be expressed as those instances where individuals, either themselves or on behalf of an organization, purposefully use ambiguity to accomplish defined goals [16,110]. Strategic ambiguity allows multiple perspectives and narratives to co-exist within the same communication, especially where certain stakeholders may have a different sense of what they believe to be truth [16,111]. This means that considering only disclosed one-way communication is of limited value in creating a consensus approach to animal welfare narratives, arguments, and discourse and ultimately developing and embedding good welfare policies and practices. The degree of transparency therefore is an important aspect to consider when reflecting on publicly disclosed information. 

Transparency is the information about decisions and decision-making processes that is provided or made available to the public, and as such can be seen to be either partial or full transparency or disclosure [112,113]. Supply chain transparency initiatives, such as the Better Chicken Commitment, can increase consumer/stakeholder pressure on corporates to disclose information [114] and make promises for the future. Transparency in this context describes the visibility and accessibility of data associated with production processes and the associated disclosure activities by one actor to other actors in the supply chain [16,113,115]. Failure to disclose information, or perception of failure to disclose information to others creates a communication gap and in this situation the resultant opacity can create perceptions of weak corporate integrity or lead to a failure to fully engage consumers in ethical forms of consumerism to drive change supply chain practice.

Ethical consumerism is the conscious and deliberate choice to make certain consumption decisions based on personal and moral beliefs [116,117], or alternatively, ethical consumerism has been described as the “political, religious, spiritual, environmental, social or other motivations for choosing one product over another.” [118] (p. 2). More simply, ethical consumption requires consumers to reduce cognitive dissonance and consider the effects of their food choice on issues such as animal welfare and decide whether they wish to use their purchasing power to influence corporate practice [119,120]. Ethical consumerism can drive supermarket strategies, i.e., “the techniques and systems in place that retailers use to effectively market their products to consumers” [121] (p. 147). These strategies can be enforced within the store or by supply chain approaches that drive higher animal welfare standards, better resource use or a reduction in product-related environmental footprint. However, enabling stakeholder participation through inclusive and collective dialogues still proves challenging.

### 3.2. Developing Collective Dialogue

Mullan et al. [98] proposes four solutions to difficulties that may arise when developing a collective and co-owned stakeholders’ dialogue. These include allowing sufficient time for key stakeholders’ dialogues to form, ensuring involvement of all interested parties in the creation and maintenance of shared dialogue, using facilitation techniques to engage and include, and also distinguishing clearly between experimental and applied science when developing science-based discourse. Using a multi-actor approach to explore values, preferences, expectations and risk perceptions of multiple stakeholders such as farmers, food companies and retail businesses can facilitate engagement to co-create knowledge and achieve mutual goals [122], such as with the Better Chicken Commitment. 

The evidence of collective dialogue in animal welfare can be clearly seen in the case of welfare assessment protocols [123,124]; religious slaughter [124]; and animal behavior [24]. By way of illustration, Ref. [88] describes the methods for establishing successful science and societal dialogue for the Welfare Quality^®^ assessment protocol. To design the framework, there were numerous interactions between animal scientists, social scientists, and members of the public. The social construction process was achieved through multi-actor interactions included meetings, conferences, workshops, websites, newsletters, interviews, focus groups, and citizen and farmers juries. Through these series of collaborations, the stakeholders developed twelve welfare criteria, which were vetted by citizen juries. Animal scientists therefore took account of societal opinion when developing the farm animal welfare assessment tools which can increase the likelihood of palatability of use and outcomes for societal expectations and animal welfare standards on farm. 

Dialogue between scientists and farmers can be complex to facilitate and can have repercussions for the scientific-societal relationship [125]. This study found that farmers were only moderately open to scientific knowledge on animal behavior relating to reducing tail biting in pigs, and found scientific solutions proposed to be too uncertain, not well understood or not applicable in their context. The authors did however find that dialogues between scientists and farmers did lead to improved mutual trust and understanding of each other’s framing and context. Both groups appeared to react and argue from their praxis, including their local environment and situation, especially their way of living and understanding their environment. Therefore, stepping into each other’s praxis through social learning might provide the concrete and fusing insights, required to facilitate joint co-constructed learning processes [24,125]. The challenges facing animal welfare scientists and society are rooted in the trade-offs between perceptions of animals as production units and ethical positioning of animals as sentient beings. Sandøe et al., [126] identified four key value-related questions that have troubled the scientific definitions of animal welfare namely: What is the benchmark for morally acceptable animal welfare? What is a good animal life? What farming purposes are legitimate? And. What kinds of compromise are acceptable in a less-than-perfect world? These are legitimate questions, and this research proposes that animal welfare scientists should consider them carefully when developing welfare assessments that are properly interpreted and applied to practical situations. Such reflection will lead to a more transparent appreciation of the values underlying welfare assessment [126].

The concept of positive welfare is being proposed by the literature as a way of shifting the definition of animal welfare from reduction in negative experiences towards enhancing positive welfare experiences and towards a good life [61]. A person’s way of conceptualizing and studying positive welfare is linked therefore to their own ethical beliefs [127]. This difference in perceptions is responsible for multiple co-existing concepts and definitions of welfare that are influenced by ethical views from different interest groups including the scientific community [127]. Therefore, multiple perceptions of good welfare through to a good life [30] can exist without necessarily conflicting with one another based on value-based judgments of what positive welfare is. Positive welfare acknowledges sentience and anthropomorphic terms (such as happy, pleasure) which demonstrate advancement in the animal welfare research domain. Nevertheless, a collective dialogue and co-created definition of “positive welfare” which allows for dynamic progression of research and translating this to farmer led innovation is necessary for extant science to make a meaningful contribution to society [127]. 

**Figure 1:**
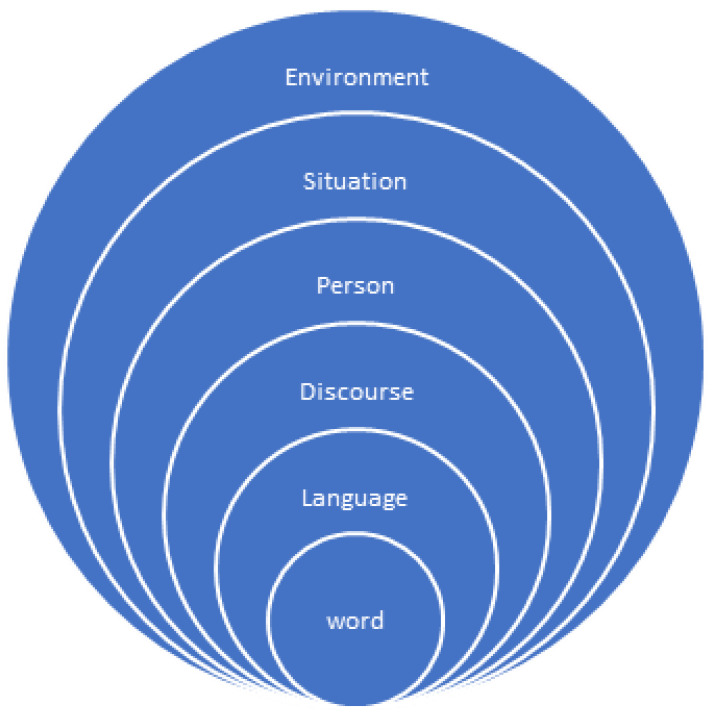
Animal welfare discourse explanatory framework Adapted from [23].

In reflecting on the animal welfare discourses within this review and the particular focus on content and context, an explanatory framework has been developed to inform future work on animal welfare discourse analysis. Previous work by [23], who considered the word (s), the discourse and the context, can inform how we interpret discourse. Figure 1 presents an explanatory framework that builds on this work in terms of word-language-discourse-person-situation-environment dynamic that can be used to evaluate and interpret the structure of single and multi-stakeholder communications. This captures the themes running through this review paper.

At the first level words such as welfare, husbandry and farming are used. These are then framed into language. The language used then implicitly or explicitly infers particular meaning. In the context of welfare, the language used could be good or bad welfare, positive or negative welfare. Similarly, the word farming has been extended in narratives to language such as welfare friendly farming, sustainable farming, factory farming, intensive farming or extensive farming. The language is structured into a given discourse. The person, their positionality, role or identity also frame the discourse, e.g., being the regulator, the vet, the farmer, or the consumer and this can have both implicit and explicit impact on the words and language used. In future animal discourse or dialetic studies, the critical discourse analysis needs to move beyond content analysis (the words and language) and the themes that can arise which can be associated with given meanings. This means beyond the person the situation and the socio-economic and socio-political environment needs to be considered and how this influences language, discourse and the meanings that are derived. The explanatory framework herein can support qualitative research and criticality in discourse analysis. The framework informs consideration of narratives and co-created and contested discourse among and between stakeholders.

## 4. Conclusions

This review has explored the contribution of narrative and argumentative discourse to the social construction and framing of animal welfare. Stakeholder discourse is contested, as is the positioning of a good life for farm animals which is rooted in multiple utility based or values-laden narratives. To resolve these differences and advance animal welfare research and implementation in practice, developing a collective understanding and collective narrative among stakeholders is crucial. The explanatory framework described in this paper can potentially be a useful tool to achieve this outcome. The concept of positive welfare has a scientific and societal appeal and can prove to be a useful mechanism in advancing farm animal welfare. Positive welfare as a concept, challenges contemporary use of animal welfare related language, understandings and attitudes and requires farm practices to be adapted to go beyond minimizing negative experiences and proactively promoting positive experiences for farm animals too. Therefore, future research should critically examine both the dialogues and discourses expressed among and between stakeholders to embody the breadth, depth, and understanding of the multiple meanings of farm animal welfare, through the emergent positioning of positive welfare for farm animals as well as how to achieve good welfare in practice. Further research on the articulation and implementation of discourses that support the social construction of collective narratives within the farm animal welfare debate would also be of value.

## Figures and Tables

**Table 1:** Characteristics of dialogues.

Characteristic	Source
Collaborative, cogenerative, consensus driven	[6,7,8]
Constructive	[8]
Internal or external	[12]
Intensified	[4]
Institutionalised	[5]
Mutually beneficial	[9]
Persuasive	[11]
Pluralistic	[10]
Surface/deep	[13]
Synchronous/asynchronous	[11]

## Data Availability

Not applicable.
